# Draft Genome Sequence of the Plant Growth-Promoting Rhizobacterium *Klebsiella* sp. Strain KBG6.2, Imparting Salt Tolerance to Rice

**DOI:** 10.1128/MRA.00491-20

**Published:** 2020-08-27

**Authors:** Berhanu Girma, Ananta N. Panda, Sasmita Mohanty, Lopamudra Ray, Gopal Chowdhary

**Affiliations:** a Plant Molecular Biology Laboratory, School of Biotechnology, KIIT Deemed to be University, Bhubaneswar, Odisha, India; b College of Agriculture, Wollo University, Dessie, Amhara, Ethiopia; c Environmental Biotechnology Laboratory, School of Biotechnology, KIIT Deemed to be University, Bhubaneswar, Odisha, India; d Department of Biotechnology, Rama Devi University, Bhubaneswar, Odisha, India; e School of Law, KIIT Deemed to be University, Bhubaneswar, Odisha, India; University of Arizona

## Abstract

*Klebsiella* sp. strain KBG6.2 is a potential salt-tolerant, plant growth-promoting rhizobacterium isolated from a rice field in Konark, Odisha, India. Here, we report the whole-genome sequencing of *Klebsiella* sp. strain KBG6.2, which has a 5.038-Mb genome containing 4,867 predicted protein-coding sequences and 79 RNA genes.

## ANNOUNCEMENT

In recent years, plant growth-promoting rhizobacteria (PGPR) have emerged as a potential tool to counter abiotic stresses (1), an important alternative to chemical fertilizer for sustainable agricultural production, and a means to promote plant growth under adverse environmental conditions. Some strains within the *Klebsiella* bacterial group, such as *Klebsiella* sp. strain IG3, have already been documented for their potential in promoting plant growth and imparting significant salt tolerance to oat plants (2). Soil samples were obtained from an agricultural field in Konark (19°53ʹ14.80ʺN, 86°05ʹ40.55ʺE), Odisha, India. Konark is a coastal area, and about 47.2% of the area is covered under various cropping systems. Because it is a coastal area, the salt concentration is comparatively higher than the global average of salt concentrations in irrigated soil (3); this has a negative effect on the health and growth of crop plants. Here, the investigators have made an attempt to isolate and to characterize PGPR imparting salt tolerance to rice plants. The soil samples were serially diluted to 10^−8^ using sterile 0.9% sodium chloride and plated onto LB agar medium, followed by incubation at 37°C for 24 h. The bacteria were screened based on size, morphology, and colony type, followed by subculturing to obtain pure colonies. The isolate *Klebsiella* sp. strain KBG6.2 was evaluated for its salt tolerance by growth on LB broth supplemented with sodium chloride.

The isolate *Klebsiella* sp. KBG6.2 was cultured and maintained in LB medium (agar and broth) and was stored as glycerol stocks at −80°C. The genomic DNA was extracted from a pure culture grown in LB broth using a genomic DNA isolation kit (MP Biomedicals, Santa Ana, CA). A DNA library was prepared using the NEBNext Ultra DNA library preparation kit according to the manufacturer’s manual. The whole-genome sequencing of *Klebsiella* sp. KBG6.2 was performed using the Illumina HiSeq X Ten system (AgriGenome, SciGenom Labs Pvt. Ltd., Kerala, India), producing a total of 18,066,246 bp reads with 2 × 150-bp read chemistry and giving a coverage of ∼606-fold. *De novo* assembly was performed using the Unicycler (version 0.4.8) assembler (4) with default parameters.

*Klebsiella* sp. KBG6.2 was found to be able to grow in 24% (wt/vol) sodium chloride, which is significantly higher than its previously known salt-tolerant sibling *Klebsiella* sp. IG3, which could tolerate up to only 20% salt (2). The whole-genome sequencing data were uploaded to the Rapid Annotations using Subsystems Technology (RAST) server for genome annotation, and the Prokaryotic Genome Annotation Pipeline (PGAP) was used for the prediction of protein-coding genes and the design of functional genome units such as RNAs, tRNAs, and small-subunit rRNAs (5). The distribution of various coding sequences (CDSs) and BLAST results with the closest species type strains were plotted using the CGView server for circular genome visualization. A comparative genome study of *Klebsiella* sp. KBG6.2 was performed with available genomes of the closest species type strains, based on 16S rRNA sequence identity, DNA-DNA hybridization (DDH), and difference in G+C contents.

The complete genome sequence analysis of the raw reads obtained from the Illumina HiSeq X Ten platform resulted in 5.03 Mb split across 54 contigs and a G+C content of 57.7%. The size of the largest contig was 1.278 Mb, and the *N*_50_ value was found to be 293,194 bp. Upon annotation of the whole genome of the strain, *Klebsiella* sp. KBG6.2 was found to contain 4,812 predicted CDSs. Of these, 4,763 predicted genes had functional annotation and 79 genes were for RNAs, including 76 tRNAs and 3 rRNAs (1 each of 23S rRNA, 16S rRNA, and 5S rRNA). Further, a total of 3,167 genes were predicted to be involved in 230 functional pathway categories, and some PGPR-related genes were identified based on the Kyoto Encyclopedia of Genes and Genomes (KEGG) pathway database via the KEGG Automatic Annotation Server (KASS). The majority of genes annotated via the KASS were predicted to be involved in various metabolic pathways; however, 95 and 54 genes were predicted to be involved in stress response and pathogen defense, respectively.

The two closest type strains, namely, Klebsiella pneumoniae subsp. *ozaenae* NCTC5050^T^ and Klebsiella variicola DSM 15968^T^, were chosen for the comparative genome analysis (Table 1). *Klebsiella* sp. KBG6.2 showed 16S rRNA similarity of 99.72% with both Klebsiella pneumoniae subsp. *ozaenae* NCTC5050^T^ (GenBank accession no. UGLZ00000000) and Klebsiella variicola DSM 15968^T^ (NZ_CP010523) and DDH similarity values of 84.2% and 74.87%, respectively. However, Klebsiella variicola DSM 15968^T^ and Klebsiella pneumoniae subsp. *ozaenae* NCTC5050^T^ had genome sizes of 5.52 Mb and 5.358 Mb, respectively, while *Klebsiella* sp. KBG6.2 had a genome size of 5.038 Mb.

**TABLE 1:** Comparison of *Klebsiella* sp. KBG6.2 with available genomes of its closest neighbors

Reference genome species^*a*^	Strain	GenBank accession no.	DDH (%)	G+C content difference (%)	16S rRNA similarity (%)
*Klebsiella variicola*	DSM 15968^T^	NZ_CP010523.2	74.87	0.17	99.72
*Klebsiella pneumoniae* subsp. *ozaenae*	NCTC5050^T^	UGLZ00000000.1	84.2	0.69	99.72
*Klebsiella quasipneumoniae* subsp. *quasipneumoniae*	01A030^T^	CCDF00000000.1	70.43	0.24	99.65
*Klebsiella quasivariicola*	KPN1705^T^	NZ_CP022823.1	66.37	1.07	99.59
*Klebsiella pneumoniae* subsp. *pneumoniae*	DSM 30104^T^	AJJI00000000	88.9	0.71	99.52
*Klebsiella pneumonia* subsp. *rhinoscleromatis*	ATCC 13884^T^	ACZD00000000.1	87.63	0.5	99.52
*Klebsiella quasipneumoniae* subsp. *similipneumoniae*	07A044^T^	CBZR00000000.1	74.53	0.48	99.45
*Enterobacter cloacae* subsp. *dissolvens*	ATCC 23373^T^	WJWQ00000000.1	25.87	2.56	98.83
*Enterobacter aerogenes*	KCTC 2190^T^	CP002824.1	41.73	2.88	98.76
*Enterobacter cloacae* subsp. *cloacae*	ATCC 13047^T^	NC_014121.1	24.87	3.14	98.49

a The two closest strains found were Klebsiella pneumoniae subsp. *ozaenae* NCTC5050^T^ and Klebsiella variicola DSM 15968^T^, with 16S rRNA similarity of 99.72%.

The genome of *Klebsiella* sp. KBG6.2 contained genes for ammonia production (*ureF*, *ureG*, *ureE*, and *ureD*), phosphate solubilization (*pqqD*), siderophore synthesis (*entB*), receptors (*dnaJ* and *tonB*), ferric uptake regulation (*fur*), and heat (*groEL* and *groES*) and cold (*cspA* and *cspC*) shock responses, as well as genes for resistance to heavy metals (*copD*, *rcnA*, and *czcD*). These groups of genes may be facilitating and are probably responsible for acclimation of plant seedlings to stress conditions, as shown in *Bacillus* sp. strain JS and *Klebsiella* sp. strain D5A (6, 7). *Klebsiella* sp. KBG6.2 needs further molecular and biochemical characterization to determine its potential role in abiotic stress tolerance and allied signal transduction processes.

### Data availability.

The whole-genome shotgun project for *Klebsiella* sp. KBG6.2 has been deposited in DDBJ/ENA/GenBank under the accession no. WSLL00000000 and BioProject and BioSample accession no. PRJNA594627 and SAMN13523692, respectively. The version described in this paper is version WSLL01000000. The 16S rRNA nucleotide sequence of *Klebsiella* sp. KBG6.2 has been deposited in GenBank under the accession no. MN918258. The raw sequencing reads have been deposited in the NCBI SRA under the accession no. SRR12095175.
